# 二代测序检测多发性骨髓瘤IGH基因克隆性重排临床价值

**DOI:** 10.3760/cma.j.issn.0253-2727.2021.08.013

**Published:** 2021-08

**Authors:** 利 姚, 艳 陈, 英颖 翟, 晓兰 施, 建农 岑, 灵芝 颜, 琤琤 傅, 苏宁 陈

**Affiliations:** 苏州大学附属第一医院，江苏省血液研究所，国家血液系统疾病临床医学研究中心，国家卫生健康委员会血栓与止血重点实验室 215006 National Clinical Research Center for Hematologic Diseases, Key Laboratory of Thrombosis and Hemostasis of Ministry of Health, Jiangsu Institute of Hematology, the First Affiliated Hospital of Soochow University, Suzhou 215006, China

多发性骨髓瘤（MM）是一种克隆性的浆细胞肿瘤，其特征为单克隆免疫球蛋白（IG）的异常浆细胞在骨髓内恶性增殖[1]。因此，判断异常浆细胞的单克隆性、克隆类型、比例以及选择合适的生物标志，对于MM的诊断及治疗随访监测至关重要[2]–[3]。在过去近20年，欧洲BIOMED-2重排检测方案已逐渐成为检测IG基因克隆性重排的金标准[4]。近几年，二代测序（NGS）技术在临床得到广泛应用[5]。本研究中，我们对初诊MM患者进行基于NGS的IGH基因克隆性重排检测，探讨其在MM中的临床价值。

## 病例与方法

1. 病例：以2019年9月至2020年10月来本院就诊的60例初诊MM患者为研究对象，其中男33例，女27例，中位年龄57（39～70）岁。以5名正常人外周血的混合样本作为对照，标本来自苏州大学附属第一医院生物样本库。诊断符合2016年IMWG MM诊断指南[2]和2020年中国MM诊治指南[3]。

2. DNA提取及鉴定：采集EDTA抗凝的患者骨髓，采用红细胞裂解液离心得到骨髓有核细胞。采用DNA提取试剂盒（美国Promega公司产品）提取DNA。采用分光光度计（美国Thermo公司产品）检测DNA浓度与纯度。采用Qubit（美国Thermo公司产品）定量待测标本的DNA浓度。

3. 文库制备和上机：NGS采用扩增子法建库方法来检测IGH基因重排克隆。按照LymphoTrack™重排NGS检测试剂盒（美国Invivoscribe公司产品）说明书进行操作，对患者标本进行IGH-FR1、IGH-FR2和IGH-FR3基因重排检测。PCR条件：95 °C预变性7 min，95 °C 45 s、60 °C 45 s、72 °C 90 s，共29个循环，最后72 °C延伸10 min。采用定量PCR检测试剂盒Ion Library^®^ TaqMan Quantitation Kit（美国Applied Biosystems公司产品）对文库定量，随后进行油包水和文库富集。采用Ion S5测序仪（美国Thermo Fisher公司产品）对富集文库进行测序。

4. PCR结合毛细管电泳法（CE）：按照IdentiClone™重排试剂盒（美国Invivoscribe公司产品）说明书进行操作，对患者标本PCR扩增IGH-FR1、IGH-FR2和IGH-FR3基因重排。PCR条件：95 °C预变性7 min，95 °C 45 s、60 °C 45 s、72 °C 90 s，共35个循环，最后72 °C延伸10 min。取PCR扩增产物1 µl与10 µl甲酰胺、0.1 µl GeneScan-500 LIZ（美国Applied Biosystems公司产品）混合，经95 °C 2 min热变性，4 °C保存。采用3730基因分析仪（美国Applied Biosystems公司产品）进行PCR片段分析，判断标准见参考文献[4]。

5. 数据分析和统计学处理：NGS测序数据使用商品化试剂配套的一站式自动化数据分析流程软件对序列进行比对分析。统计学分析采用SPSS10.0软件，分类变量组间比较采用Fisher精确概率法，连续变量组间比较采用Mann-Whitney *U*检验，*P*<0.05为差异有统计学意义。

## 结果

1. DNA质量检测：本组样本DNA浓度均大于50 ng/µl，DNA纯度为1.8～1.9。DNA琼脂糖电泳检测显示DNA完整性好，无DNA降解断裂情况。

2. NGS和CE法的比较：60例初诊患者同时用NGS和CE检测IGH基因重排克隆。两种方法IGH基因克隆性重排双阳性42例，双阴性17例，其中IGH-FR1检测的一致性为91.7％（55/60），IGH-FR2检测的一致性为93.3％（56/60），IGH-FR3检测的一致性为96.7％（58/60），IGH检测方法总体的一致性为98.3％（59/60）。

3. NGS-IGH基因重排分析：60例MM患者中IGH-FR1重排阳性37例，阴性23例，检出率为61.7％ ；IGH-FR2重排阳性38例，阴性22例，检出率为63.3％；IGH-FR3重排阳性29例，阴性31例，检出率为48.3％。联合使用IGH-FR1/FR2/FR3重排的总体检出率为70.0％（42/60）（表1）。

**表1:** 初诊多发性骨髓瘤患者中IGH各区域的克隆性重排检出率［例（％）］

IGH重排	FR1	FR2	FR3	FR1/FR2/FR3
克隆性重排	37（61.7）	38（63.3）	29（48.3）	42（70.0）
非克隆性重排	23（38.3）	22（36.7）	31（51.7）	18（30.0）

4. IGH基因克隆性重排患者临床特征：有无IGH基因克隆性重排初诊MM患者临床特征比较见表2，两组性别、年龄差异无统计学意义。经免疫蛋白固定电泳检测M蛋白类型，其中IgG型28例，IgA型13例，轻链型14例，IgD 3例，寡分泌型1例，不分泌型1例。比较不同组别M蛋白类型的检出率，IgG型与非IgG型的IgH基因重排阳性率之间差异有统计学意义（85.7％对56.3％，*P*＝0.028）。国际分期系统（ISS）分期中Ⅰ期21.7％、Ⅱ期45.0％、Ⅲ期33.3％，修订后的国际分期系统（R-ISS）分期中Ⅰ期15.0％、Ⅱ期68.3％、Ⅲ期16.7％，两组ISS、R-ISS分期差异无统计学意义。

**表2:** 有无IGH基因克隆性重排初诊多发性骨髓瘤患者临床特征比较

临床特征	IGH重排组（42例）	非IGH重排组（18例）	*P*值
性别（例）			0.533
男	22	11	
女	20	7	
中位年龄（岁）	57（39～70）	54（43～66）	0.890
M蛋白类型（例）			0.019
IgG型	24	4	0.028
非IgG型	18	14	
ISS分期（例）			0.062
Ⅰ期	8	5	
Ⅱ期	23	4	
Ⅲ期	11	9	
R-ISS分期（例）			0.371
Ⅰ期	5	4	
Ⅱ期	31	10	
Ⅲ期	6	4	

注：ISS：国际分期系统；R-ISS：修订后的国际分期系统

5. 重排克隆类型和比例：在42例显示克隆性IGH基因重排的样本中，其中41例均检测到产物性重排，1例仅检测到非产物性重排。在IGH基因重排中，可变区（V区）取用频率最高的为IGHV1-18、IGHV3-23、IGHV3-30、IGHV4-39，分别占8.9％，其次为IGHV4-59和IGHV5-51，分别占6.7％。多样区（D区）取用频率最高的为IGHD3和IGHD2，分别占33.3％和24.4％；其中IGHD2-2、IGHD2-21分别占11.1％，IGHD4-17、IGHD3-3、IGHD3-16、IGHD3-10分别占8.9％。连接区（J区）取用频率最高的为IGHJ4和IGHJ6，分别占40.0％（图1）。1例患者同时检测到IGHV4-59/J6和IGHV3-33/J6两种类型的产物性重排。3例患者同时检测到1种类型的产物性重排和1种类型的非产物性重排，分别为IGHV5-51/J4和IGHV7-4/J1、IGHV3-30/J6和IGHV4-39/J6、IGHV2-70/J6和IGHV1-46/J4。并且42例患者均显示各自独特的IGH基因重排克隆序列。初诊患者IGH基因重排优势克隆的比例中位数为22.7％（5.4％ ～88.3％），其中V区SHM的中位突变率为9.8％（2.9％～27.1％）。

**图1:**
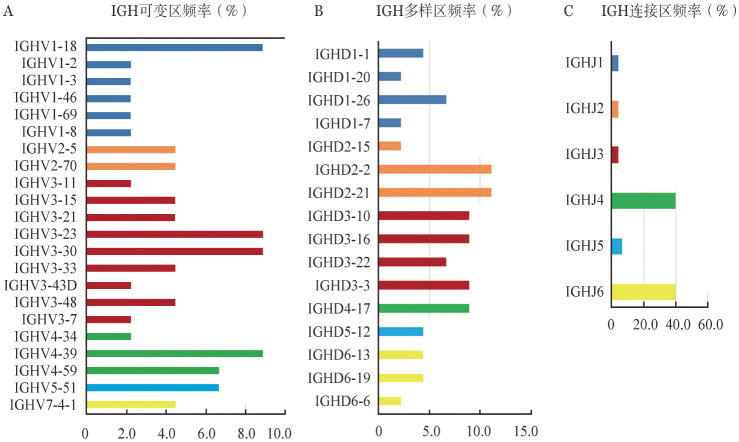
多发性骨髓瘤患者中IGH重排克隆可变区（A）、多样区（B）及连接区（C）片段的取用频率

## 讨论

在正常B淋巴细胞发育分化过程中，处于胚系状态的IGH基因的V/D/J片段均需发生基因重排。IGH基因重排先于IG轻链基因重排，首先在早前B细胞（Pro-B cell）中形成DJ连接，随后在前B细胞（Pre-B cell）中V基因片段连接到D片段上，最终形成VDJ基因重排。由于参与重排基因片段的多样性、重排随机性以及重排过程中连接区核苷酸的随机插入和删除，导致每个克隆B细胞均有各自独特的基因重排方式，可作为B淋巴细胞的克隆基因标志物[6]。

在淋巴系统肿瘤的辅助诊断中，欧洲BIOMED-2重排检测方案已逐渐成为IG基因克隆性重排检测的金标准[4]。但该方案也有自身的局限性，如只能对目的片段进行定性，不能检测重排克隆的具体类型和比例。而NGS方法是在BIOMED-2的基础上，采用扩增子法建库，并通过对文库进行测序来识别各种不同的重排克隆序列，实现了克隆序列的准确识别和定量[7]。本研究我们将NGS与CE方法进行比较，结果显示两种检测方法具有相似的检出率，IGH基因检测的总体一致性为98.3％。同时NGS结果显示，在IGH-FR1、IGH-FR2和IGH-FR3克隆性重排中都有较高的检出率，频率大小依次为IGH-FR2、IGH-FR1、IGH-FR3，联合应用IGH-FR1/FR2/FR3的总体检出率为70.0％。并且42例初诊患者均显示各自独特的IGH基因重排克隆序列，重排优势克隆的比例为22.7％（5.4％～88.3％）。

本组MM患者的临床特征与IGH基因重排之间的关系显示，IGH基因克隆性重排与患者性别、ISS分期差异无统计学意义，但与M蛋白类型相关。本组患者M蛋白类型以IgG型最多，IgA和轻链型其次，其分布类型与国内文献报道相符[8]。有意思的是，本研究发现，IgG型MM患者的IgH重排阳性率（85.7％，24/28）高于非IgG型（56.3％，18/32），差异有统计学意义（*P*＝0.028），提示IGH基因重排克隆与M蛋白类型之间存在相关性。IGH基因克隆性重排主要存在于重链型中，而单纯轻链型中较少发生。基因重排克隆在不同类型MM患者中可能存在不同的重排发生机制，从而分泌各种异常的免疫球蛋白类型，具体发生机制有待更深入的研究。

在B细胞的成熟过程中，IG分子还要经过生发中心的体细胞超突变（SHM）才能完成抗体亲和成熟。慢性淋巴细胞白血病（CLL）患者IGH基因V区SHM检测已明确对疾病预后相关。本研究结果显示在MM患者的浆细胞中，IGH基因V区显示SHM突变频率为9.8％（2.9％～27.1％）。相对于其他B细胞恶性肿瘤，如CLL等[9]，MM患者IGHV的SHM平均突变频率更高，与国外文献报道相似[10]，指出更高的突变频率可能与更好的临床预后相关。

正常B细胞在发育过程中会伴随VDJ基因重排，这些重排对V、D、J基因片段的取用是按照一定的规律随机发生的，而MM患者是否有自身的特征。本研究60例初诊MM患者的NGS检测结果显示IGHV3亚组取用频率明显高于其他组别，如IGHV3-23和IGHV3-30基因（分别占8.9％）呈优势重排克隆。而通常在正常B细胞中发生的重排，如IGHV3-20和IGHV3-74，本组患者中没有检测到克隆性重排。与Medina等[10]报道的西班牙人群MM患者优势重排克隆相比较，本组检测到IGHV7-4-1重排克隆，而西班牙研究未见报道，两组MM人群中存在显著性差异。该结果提示IGH基因重排克隆在不同人种中可能存在差异。另外有意思的是，IGHV4-34在正常B细胞、急性B淋巴细胞白血病、慢性淋巴细胞白血病、套细胞淋巴瘤中发生率高[11]–[13]，但通常在正常浆细胞群体中缺失，在MM中少见，本组病例中发现1例。该结果与Medina等[10]报道相似，且本组1例为产物性重排，其具体的生物学意义有待更深入的研究。值得注意的是，本组MM患者IGHD区中IGHD2-21（11.1％）和IGHD3-10（8.9％）的取用频率明显高于健康人，与Medina等[10]报道相似，尤其是IGHD2-21可能是MM的一个独特标志。同时Medina等[10]研究也发现IGHD2和IGHD3亚组与MM患者更好的预后相关，但由于本研究纳入分析样本量较小，这些数据仍需通过进一步扩大样本量和延长随访时间来证实。

综上所述，本研究应用NGS方法可检测大部分MM患者的IGH基因克隆性重排，NGS不仅可以检测浆细胞的克隆性，更能鉴定重排克隆的具体类型和比例，因此，对于MM患者可以选择IGH基因重排克隆作为其分子生物学标志物。此外，本结果也提示MM患者的IGH基因的V、D、J基因片段取用具有一定的偏向性，呈现独特的克隆重排谱系特征，是否与MM的发病机制和预后相关，有待更多、更详细的研究数据证实。
